# Screening and genomic evaluation of keratinolytic protease producing *Chryseobacterium* sp. from tannery waste and its potential application in dehairing of goat skin

**DOI:** 10.1016/j.jgeb.2025.100458

**Published:** 2025-01-19

**Authors:** Taslima Akter, Murshed Hasan Sarkar, Shashanka Shekhar Sarker, Nourin Tarannum, Showti Raheel Naser, Sanjana Fatema Chowdhury, Sahana Parveen

**Affiliations:** a Leather Research Institute, Bangladesh Council of Scientific and Industrial Research, Savar, Dhaka 1350, Bangladesh; b Bangladesh Council of Scientific and Industrial Research Laboratories, Dhaka 1205, Bangladesh

**Keywords:** Keratinolytic protease, Gram-negative bacteria, Genomic evaluation, Protease gene, Dehairing

## Abstract

Lime and Na_2_S, used in dehairing in the tannery industry, cause the generation of toxic wastes. Ecological security and financial issues demand a look for innovative approaches to leather dehairing free from pollution. The primary goal of this investigation was to explore keratinolytic protease producing bacteria from tannery waste, their genomic evaluation and to assess their possible use in the dehairing process. A newly isolated Gram-negative bacterium (LRI-TA6) was characterized and checked for its keratinolytic protease producing ability. The strain was identified as *Chryseobacterium cucumeris* through whole genome sequencing. The genome was 4,541,898 bp in size and contain 4,426 protein coding sequences (CDS) with 36.38 % of GC content. Twenty-five open reading frames (CDS) were found as protease producing genes notably DegQ (serine protease), DegS, and ATP-dependent protease DP (EC 3.4.21.-). The isolate revealed enzyme production throughout an extended pH range of 5.0 to 8.0, and a broad temperature range of 10 °C to 38 °C. The isolate LRI-TA6 was sensitive to all five antibiotics (amoxicillin, tetracycline, gentamicin, ciprofloxacin, and vancomycin) with zone diameter ranges from 17.2 ± 0.8 to 32 ± 0.0 mm. The enzyme was shown to have 29.9 ± 6.7 and 83.6 ± 0.2 U/ml of keratinolytic and proteolytic activity, respectively. By employing crude keratinase, the removal of hair from goat skin was accomplished in 18 h. This study is the first report to isolate and characterize *C. cucumeris* from tannery waste and also the amazing 18 h dehairing capabilities and thus might be utilized for further studies towards commercial synthesis of keratinase for use in leather processing sectors.

## Highlights

• Keratinolytic protease from a Gram-negative bacterium *Chryseobacterium cucumeris*.
• Genome contains twenty-five open reading frames notably DegQ (serine protease).
• Enzyme production occurs at pH range-5.0 to 8.0, temperature range-10 °C to 38 °C.
• Possess keratinolytic and proteolytic activity of 29.9 ± 6.7 and 83.6 ± 0.2 U/ml.
• Enzymatic treatment of goat skin results in hair removal within 18 h.

## Graphical abstract

**Figure:**
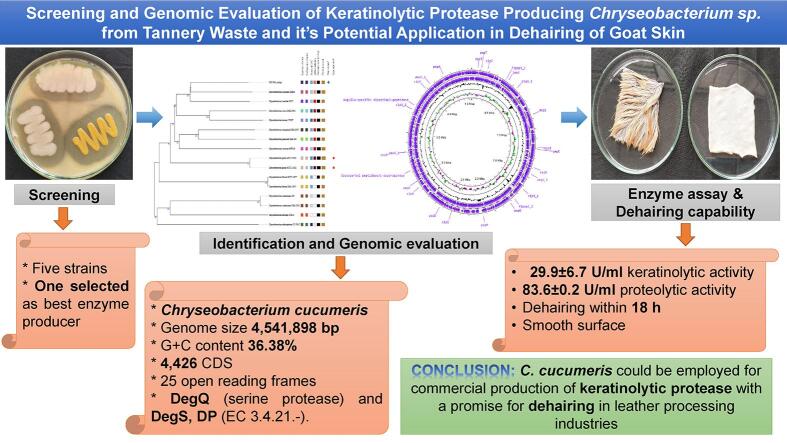

## 1 Introduction

The leather business exerts a significant induction in the world economy by providing jobs and meeting social requirements. However, the leather sector is responsible for most of the pollution of the environment.^1^ According to literature, only two to three hundred kg of leather and above six hundred kg of trash are produced from one ton of wet-salted skins.^2^ Of the various steps of leather processing, most of the wastes are generated from dehairing processes^3^ where removal of hair from hides and skins is conducted using huge amounts of lime and sodium sulfide. This results in pollution with about 60–70 % of the overall pollutant load, 100 % of the high alkaline effluent, 40 % of the BOD (biochemical oxygen demand), and 50 % of the COD (chemical oxygen demand).^4^, ^5^ Moreover, it causes a significant amount of environmental pollution^3^, ^6^ and also a decline in leather quality that damages the leather's structural integrity.^7^

To date, enzymatic dehairing is the most conducive and realistic solution to this problem for replacing harmful chemicals and reducing environmental pollution^8^ as enzymes are environmentally sustainable, non-toxic, cost-effective and have reliable process control, high yield, and also take less time to act.^9^, ^10^ Keratin has a strong, resilient matrix and the presence of large amount of disulfide bonds in its structure^11^ enables it to withstand organic solvents, mild acids and bases, water, and common proteases. Keratinases (EC: 3.4.21.11), also called keratinolytic protease, belong to a specific class of proteases, primarily serine and metalloprotease having an extended specificity of substrate that can break down hard and insoluble keratin. They are unique from other proteases due to their extended range of biochemical features and capability to break down keratin-containing substrates, including hair, wool, feathers, and nails.^12^ The efficient properties of keratinase from microorganisms of biotechnological interest align with the expanding number of industries^13^ such as textile industry, detergent industry, leather industry as well as in prion decontamination, medical and cosmetic sectors.^12^ This enzyme can convert organic wastes into bioactive peptides, valuable amino acids which could be used for the formulation of novel bio-products^14^, ^15^.

Keratinolytic proteases can be synthesized in large quantities by naturally occurring bacteria. Of these, the most active and dynamic producers are Gram-positive *Arthrobacter, Bacillus, Streptomyces, Clostridium, Actinomycetes*,^16^, ^17^ *Cellulomonas bogoriensis* ^18^ *Acinetobacter*,^19^ *Bacillus subtilis*,^20^ *Bacillus cytotoxicus* ^15^ and so on. The bacteria that are commonly utilized for keratinase synthesis on a commercial basis are *Bacillus* species.^21^ Jeong et al. ^20^ stated ninety-five species of Gram-negative *Chryseobacterium* (*Flavobacteriaceae* family). However, there are a few studies of keratinase production from Gram-negative bacteria, and not much research has been carried out on the use of *Chryseobacterium*-derived keratinase in leather processing. Based on what we know from the literature currently in circulation, this study represents a first on the generation of keratinase by the *C. cucumeris* strain isolated from precipitated dried chrome-cakes of tannery waste and also the application of this *C. cucumeris* derived keratinase in dehairing of goat skins.

## 2 Methods

### 2.1 Source of sample collection

Precipitated chrome-cakes of tannery waste were collected in a zip-lock bag (sterile) from Bangladesh Small and Cottage Industry Corporation (BSCIC) Tannery Industrial Estate at Savar in Hemayetpur, Dhaka, Bangladesh, and brought to the work place, and preserved at 4 °C until used.

### 2.2 Bacterial isolation

Serial dilution and spread plate method was employed to isolate bacteria. The dilution was conducted up to 10^−6^ fold and dispersed on nutrient agar (NA) medium. After incubation at 37 °C for 24 h, the plates were inspected for bacterial growth. Colonies with the same morphology were taken as the same bacteria and with different morphology were considered as different bacteria. The distinct colonies were then streaked on NA and incubated at 37 °C for 24 h to obtain pure colonies^22^.

### 2.3 Detection of strains for keratinolytic protease synthesis

Each single colonies were streaked on skim milk agar medium (SMA) (Hi-media, India) to find out the keratinolytic protease producing strains.^17^ After incubation for 48 h at 37 °C, the plates were inspected for the appearance of a clear zone surrounding each streak. Strains with zone diameters greater than 10 mm were elected^23^ and streaked on Casein agar (CA) medium (Hi-media, India)^24^ and incubation was done for 48 h at 37 °C. The strains with significant zone diameters were selected for further assays.

### 2.4 Analysis of strain on feather meal agar (FMA)

The strains were streaked on feather meal agar **(**FMA) and inspected after incubation at 37 °C for 48 h. The composition of feather meal agar medium was (% w/v): feather meal (1.0), NaCl and NH_4_Cl (0.05), MgCl_2_ (0.01), yeast extract (0.01), K_2_HPO_4_ (0.04), KH_2_PO_4_ (0.03) and pH 7.5.^25^

### 2.5 Identification of the most potent strain

The preliminary identification was done according to Bergey’s Manual of Determinative Bacteriology^26^ and was confirmed by whole genome sequencing.

#### 2.5.1 Morphological and biochemical analysis

For morphological analysis, the pour plate method was used. Following incubation for 24 h at 37 °C, the plates were inspected for colonial features like size, opacity, color, elevation, margin, consistency, and shape.

Gelatin, casein and starch hydrolysis, MR-VP (Methyl Red-Voges Proskauer), gram staining, indole, carbohydrate fermentation test (dextrose, sucrose, mannitol, and fructose), catalase, motility, citrate utilization, and oxidase are the biochemical tests conducted.

#### 2.5.2 Molecular identification of the elected strains

The strain was grown overnight and the DNA was retrieved utilizing the genomic DNA extraction kit in line with the guidelines from the manufacturer (QIAGEN, QIAamp DNA Mini Kit) with minor adjustments. Then Illumina RNA Prep with Enrichment (L) Tagmentation (Illumina, Catalog no. 1000000124435) was used to generate DNA libraries. After amplification, sequencing was conducted using the MiniSeq® sequencing system (Illumina). Sequence reads were assembled using the BV-BRC 3.38.9 (https://www.bv-brc.org/). The assembled genome was submitted to the comprehensive genome analysis service at PATRIC^27^. The RAST tool kit (RASTtk) was used for genome annotation^28^. FastQ sequence files were submitted to the PubMLST^29^ database for strain identification and after that a phylogenetic tree was generated in the type strain genome (tygs) server (https://tygs.dsmz.de/). Finally, the sequence was submitted to NCBI, SRA database to get the accession number for the isolate.

### 2.6 Antibiotic susceptibility pattern of the isolate

The antimicrobial sensitivity profiles of the strain for the antibiotic amoxicillin (30 µg), tetracycline (30 µg), gentamicin (10 µg), ciprofloxacin (5 µg), and vancomycin (30 µg) were evaluated following the Kirby-Bauer disk diffusion method in compliance with the breakpoints recommended by the CLSI.^30^ A variety of antibiotic classes, employed were protein synthesis inhibitors (tetracycline and gentamicin), cell wall synthesis inhibitors (amoxicillin and vancomycin), and DNA synthesis inhibitors (ciprofloxacin). The antibiotic disks were collected from Bioanalyse®.

### 2.7 Optimization of growth parameter (Temperature and pH)

A range of pH values from 4 to 10 (4, 5, 6, 7, 8, 9, 10) and a temperature ranges of 5 to 47 °C (5, 8, 11, 14, 17, 20, 23, 26, 29, 32, 35, 38, 41, 44, and 47 °C) was tested to find the ideal pH and temperature for keratinase synthesis.

### 2.8 Enzyme production and assay

#### 2.8.1 Preparation of the inoculum and keratinase production

The strain was grown for the entire night and inoculated into a medium with composition (g/l): feather meal powder (10), yeast extract (0.2), NaCl (1.0), NH_4_Cl (1.0), K_2_HPO_4_ (0.6), MgCl_2_·6H_2_O (0.5), KH_2_PO_4_ (0.8), pH (7), and 5 % (v/v) inoculum^31^. The strains were incubated for 72 h at 30 °C, and 150 rpm. Then centrifugation was conducted at 10,000 rpm for 15 min at 4 °C and the cell-free supernatant was collected and preserved for assays.

#### 2.8.2 Keratinase and protease assay

The micro-particles and bacteria were removed from the supernatant by passing across a 0.22 µm pore size filter and utilized for keratinase and protease assays. The keratinase assay was conducted following a protocol by Hossain et al.^32^ with some modifications. To sum up, 20 mg of keratin azure and 1 ml of Tris-HCl (pH 7.5, 0.05 M) was mixed and incubated at 30 °C (120 rpm) for 30 min along with 250 μl of the enzyme solution. The mixture was then neutralized with 10 % tri-chloroacetic acid (TCA) and kept at 4 °C for 30 min. After that, the supernatant was examined at 280 nm following centrifugation at 10,000 g for 10 min. In the control solution, the TCA was mixed first prior to adding the enzyme.

For protease activity, a process narrated by Keay et al.^33^ was followed with slight changes. 0.8 ml of casein solution (1 %, w/v in 0.2 mol/l tris-HCl buffer, pH 9) was mixed with 1 ml of enzyme and kept for 30 min at 30 °C. After that, 2 ml of TCA (0.4 M) was added and kept for 20 min at 30 °C and then filtered. Then, 1 ml of filtrate was mixed with 5 ml Na_2_CO_3_ (0.4 M) and 1 ml Folin & Ciocalteus phenol reagent (1:1) and kept for 20 min at 30 °C. The absorbance was taken at 660 nm with the control. In both assays, a rise in absorbance of 0.01 indicates one unit of enzyme activity.

### 2.9 Dehairing of goat skin

The goat skin was collected from the local slaughterhouse and cleaned with detergent. Then the skin was cut into pieces (2 in. × 2 in.) and weighted. Skin pieces were soaked in crude keratinase solution and kept at 30 °C. To stop bacteria from growing opportunistically, sodium azide (0.02 %) was utilized.

### 2.10 Statistical analysis

All data were taken in triplicate and all values are reported as mean ± SD. Analysis of Variance (ANOVA) was accomplished using the SPSS vs 25.0.

## 3 Results

### 3.1 Screening of potent keratinase producer

Based on the morphological variations on NA plates, ten single colonies having favorable appearance were picked. Of the ten elected strains, five, namely, LRI-TA1, LRI-TA2, LRI-TA3, LRI-TA5, and LRI-TA6 were noticed as protease producers as they form significant clear zones on SMA (Table 1) with diameter ranging from 12.7 ± 0.6 mm to 22 ± 1.0 mm.

**Table 1:** Initial screening of the strains on SMA media.

**Sl no.**	**Strain code**	**Zone of clearance (mm)**
01.	LRI-TA1	22.0 ± 1.0
02.	LRI-TA2	17.7 ± 0.6
03.	LRI-TA3	12.7 ± 0.6
04.	LRI-TA5	22.0 ± 1.0
05.	LRI-TA6	22.0 ± 1.0

Additionally, on further screening on CA media three (LRI-TA1, LRI-TA5, and LRI-TA6) out of five isolates showed significant zone of clearance (Table 2) ranging from 17 ± 1.0 mm to 22.3 ± 0.6 mm in diameter. However, isolates LRI-TA2 and LRI-TA3 didn’t show any clear zone surrounding bacterial growth. On final screening on feather meal agar (FMA), isolate LRI-TA6 showed better growth compared to LRI-TA1 and LRI-TA5.

**Table 2:** Secondary screening of the strains on CA media.

**Sl no.**	**Strain code**	**Zone of clearance (mm)**
01.	LRI-TA1	17.7 ± 1.0
02.	LRI-TA2	−
03.	LRI-TA3	−
04.	LRI-TA5	17 ± 1.0
05.	LRI-TA6	22.3 ± 0.6

**Fig. 1:**
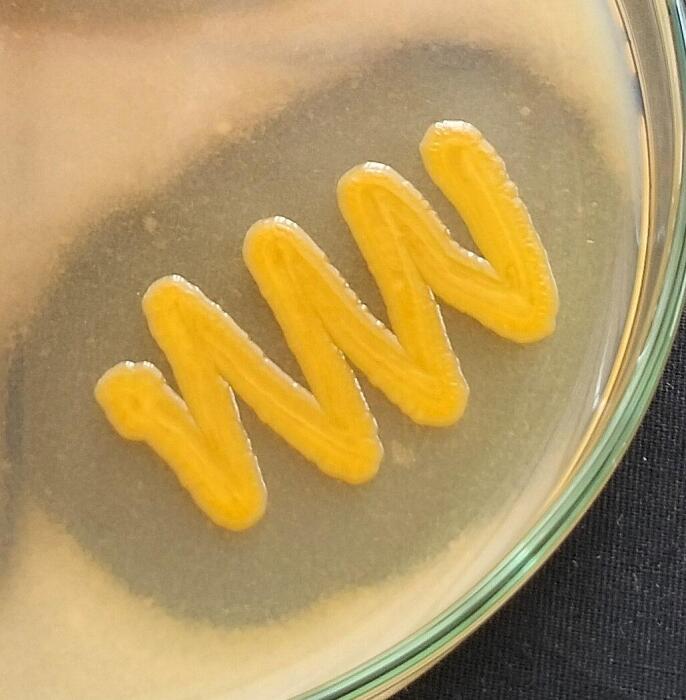

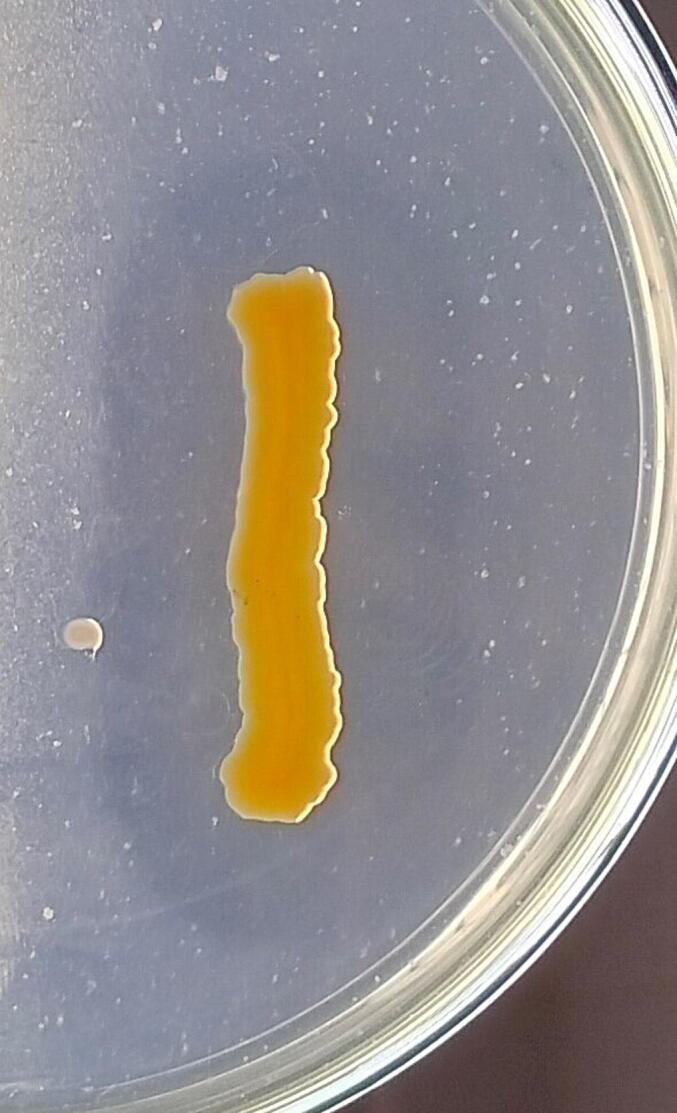

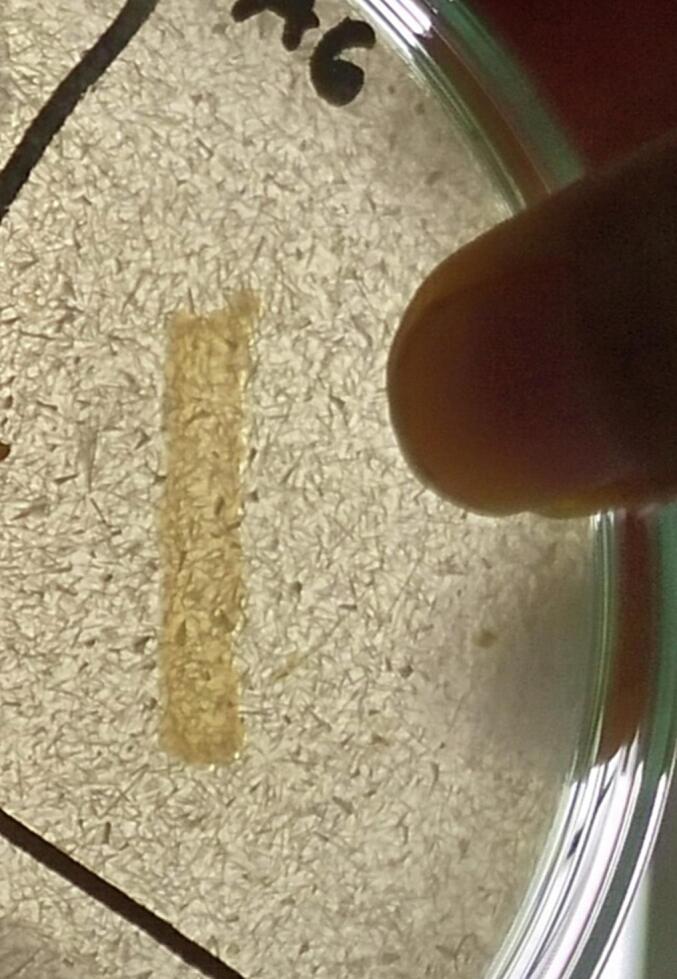
Keratinase producing activity of the isolate LRI-TA6. (a) Clearance zone on SMA (b) Clearance zone on CA (c) Growth on FMA.

Based on the highest zone diameter on both SMA (22 ± 1.0 mm) and CA media (22.3 ± 0.6 mm) and also better growth on FMA as shown in Fig. 1, the isolate LRI-TA6 was selected for further assays.

### 3.2 Morphological and biochemical features of the strain LRI-TA6

Both morphological and biochemical aspects of the selected strain LRI-TA6 were determined. The colony of the bacterium was yellowish orange in color, small circular of about 3 mm in size, entire margin and microscopically Gram-negative, non-motile, mucoid, and opaque (Table 3). The isolate showed weakly positive fermentation activity to the carbohydrates dextrose, sucrose, mannitol, and fructose; catalase, oxidase, indole and MR-VP positive; citrate negative; positive in casein and starch hydrolysis, negative in gelatin hydrolysis as shown in Table 3.

**Table 3:** Morphological and biochemical features of the strain LRI-TA6.

**Features**	**LRI-TA6**
**Morphological Tests**	
Colony size	3 mm
Color	Yellowish orange
Elevation	Slightly raised
Colony shape	Circular
Gram’s nature	Gram negative
Motility	Non-motile
Margin	Entire
Consistency	Mucoid
Opacity	opaque
**Biochemical tests**	
Carbohydrate fermentation	
Dextrose	±
Fructose	±
Mannitol	±
Sucrose	±
Catalase	+
Oxidase	+
Indole	+
MR	+
VP	+
Citrate	−
Motility	−
**Hydrolysis tests**	
Gelatin	−
Starch	+
Casein	+

Abbreviations: MR, methyl-red; VP, Voges-Proskauer.

+, −, and ± indicates positive, negative and weakly positive result.

### 3.3 Whole genome information of the isolate LRI-TA6

When compared to other genomes in PATRIC belonging to the same species and the annotation statistics, this genome seemed to be of good quality. The output of the analysis are given below.

#### 3.3.1 Assembly of the genome

An assembled genome was submitted to the Comprehensive Genome Analysis service. This assembled genome had 191 contigs, with the total length of 4,541,898 bp and an average GC content of 36.38 %. This genome has 4,426 protein coding sequences (CDS), 49 tRNA genes, and 3 rRNA genes. Table 4 provides an overview of the annotated features.

**Table 4:** The specifics of genome assembly.

Genome Length	4,541,898 bp
Contigs	191
Contig L50	32
Contig N50	40,174
GC Content	36.38
CDS	4,426
tRNA	49
rRNA	3

#### 3.3.2 Analysis of the phylogeny

**Fig. 2:**
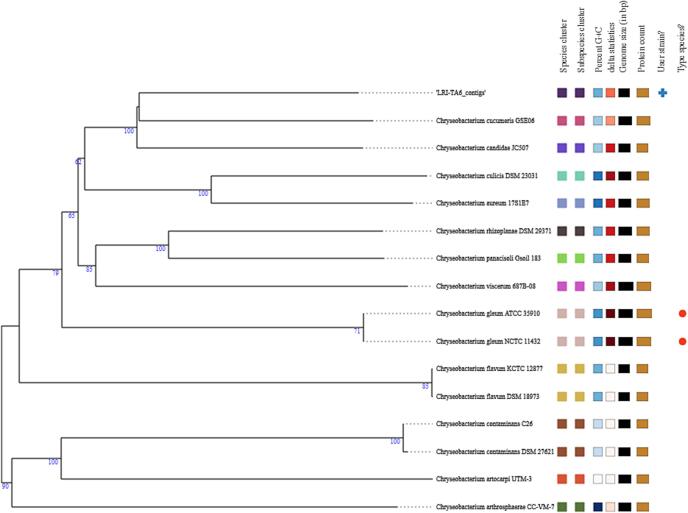
Phylogenetic tree of the study isolate LRI-TA6. The evolutionary relationships between LRI-TA6 and closely related species are displayed in this tree.

The representative and reference genomes were provided by PATRIC and were incorporated into the phylogenetic analysis included in the Comprehensive Genome Analysis report. Mash/MinHash was used to identify the closest representative and reference genomes^34^. In order to ascertain the phylogenetic position of this genome, PATRIC global protein families (PGFams)^35^ were chosen from among these genomes. MUSCLE was used to link the protein sequences of these families^36^ and for every sequence, the nucleotides were mapped to the protein alignment. After concatenating the combined set of nucleotide and amino acid alignments, a data matrix was produced which was then analyzed by RaxML,^37^ the support values in the tree (Fig. 5) were produced by employing fast bootstrapping.^38^ The nucleotide FastQ showed 100 % sequence homology and a close clustering of study strain LRI-TA6 to the *Chryseobacterium cucumeris* strain (Fig. 2).

#### 3.3.3 Genome annotation

There were 2332 proteins with functional assignments, 2094 hypothetical proteins, (Table 5), 836 proteins with Enzyme Commission (EC) numbers,^39^ 721 with Gene Ontology (GO) assignments,^40^ and 633 proteins that were mapped to KEGG pathways.^41^ Two types of protein families were annotated by PATRIC,^35^ of which one has 3725 proteins belonging to the genus-specific protein families (PLFams), and another one has 3863 proteins belonging to the cross-genus protein families (PGFams).

**Table 5:** Features of the protein.

Proteins with functional assignments	2,332
Hypothetical proteins	2,094
Proteins with Pathway assignments	633
Proteins with GO assignments	721
Proteins with EC number assignments	836
Proteins with PATRIC genus-specific family (PLfam) assignments	3,725
Proteins with PATRIC cross-genus family (PGfam) assignments	3,863

**Fig. 3:**
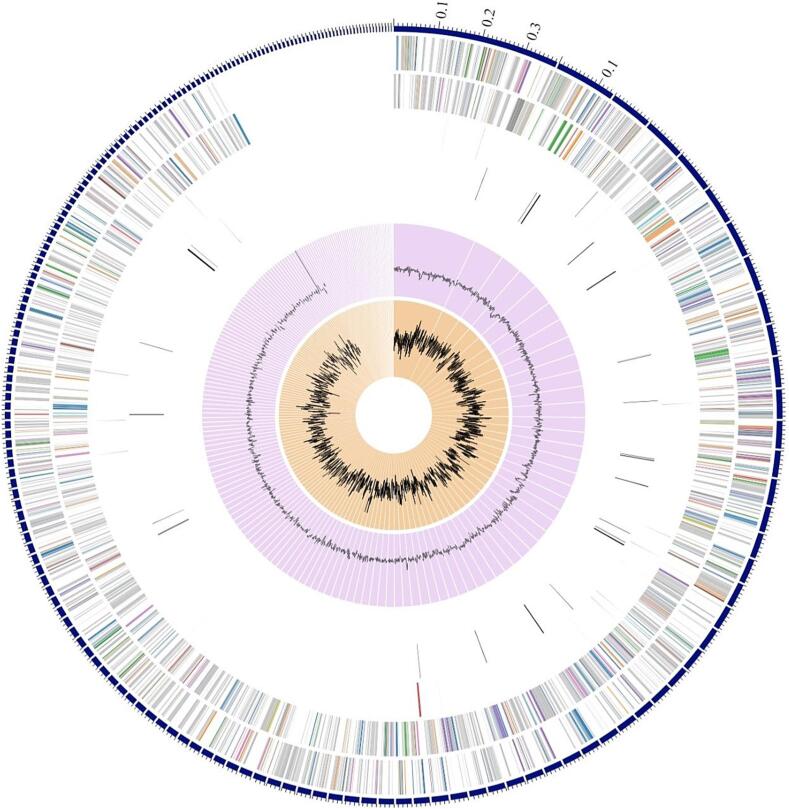
Complete map of the genome of LRI-TA6. This comprises, from outer to inner rings, the contigs, CDS on the forward strand, CDS on the reverse strand, RNA genes, CDS with similarity to known antimicrobial resistance genes, CDS with homology to know virulence factors, GC content and GC skew. The subsystem to which these genes belong is indicated by the colors of the CDS on the forward and reverse strands.

Fig. 3 shows a circular graphical representation of the genome annotation distribution. The outermost circle represents the size of the genome, whereas the genes on the positive and negative chains of the genome are represented by the second and third circles, respectively. The COG functional classifications are represented by different colors; repeat sequences are represented by the fourth circle, and tRNA and rRNA are represented by the fifth circle.

#### 3.3.4 Subsystem analysis

**Fig. 4:**
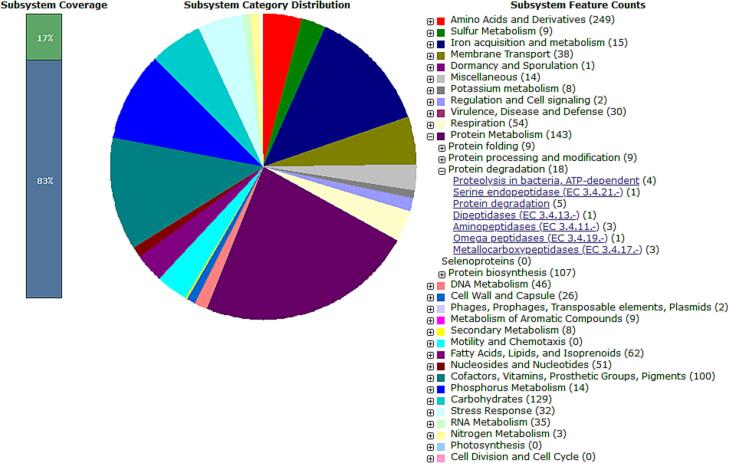
The LRI-TA6 whole-genome sequence's protein-encoding genes (pegs) were analyzed and classified into subsystems based on the RAST version 2.0 server. Leftmost bar shows percentage of pegs allocated to subsystems (green) and percentage of pegs not able to be placed into any subsystem (blue). The distribution of subsystem categories is shown in the central pie graphic. On the right, the subsystem feature counts are displayed as colored categories. (For interpretation of the references to color in this figure legend, the reader is referred to the web version of this article.)

Fig. 4 gives a summary of the subsystems for the LRI-TA6 genome.

**Fig. 5:**
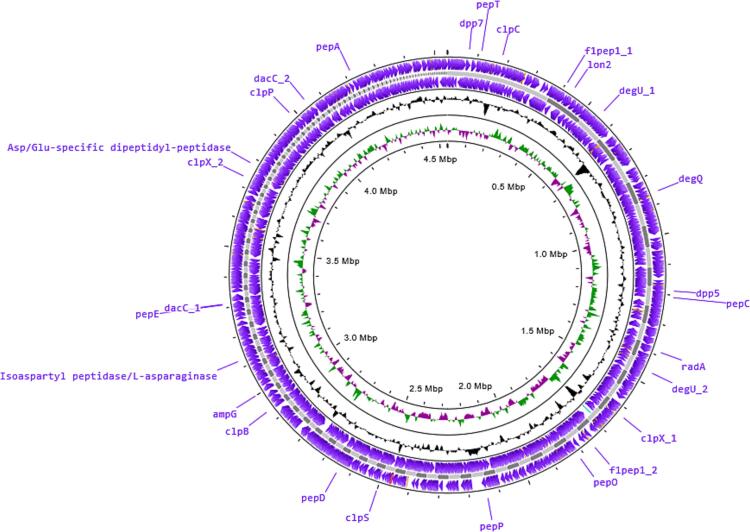
Location of protein degrading enzyme producing genes in the genome of LRI-TA6. On the right, the subsystem characteristic is indicated by the colored categories.

#### 3.3.5 Protein degrading enzyme gene analysis

A total of 25 open reading frames that produce enzymes that degrade proteins were found to be present in the CDSs as shown in Table 6 and Fig. 5, such as those for outer membrane stress sensor protease DegQ (serine protease) and DegS, ATP-dependent protease domain protein DP (EC 3.4.21.-), ATP-dependent Clp protease (ClpA, ClpC, ClpP, ClpS, ClpX, ClpQ), ATP-dependent protease (HslV, LonII), Aminopeptidase (AmpS, AmpY, IsoAsp-, PepA, PepN, PepP), ClpXP protease specificity-enhancing factor, and carboxypeptidase (DacA, Zn-Dac). The genes responsible for protease enzyme production are given in Table 6 and Fig. 5. An accession number for the strain LRI-TA6 was obtained upon submission to NCBI, SRA database and the accession number is PRJNA1179663.

**Table 6:** Protease producing genes.

**Gene**	**Name of enzyme**
AmpS	Aminopeptidase S (Leu, Val, Phe, Tyr preference) (EC 3.4.11.24)
AmpY	Aminopeptidase Y (Arg, Lys, Leu preference) (EC 3.4.11.15)
ClpA	ATP-dependent Clp protease ATP-binding subunit ClpA
ClpB	ClpB protein
ClpC	ATP-dependent Clp protease, ATP-binding subunit ClpC
ClpP	ATP-dependent Clp protease proteolytic subunit (EC 3.4.21.92)
ClpQ	ATP-dependent protease HslV (EC 3.4.25.-)
ClpS	ATP-dependent Clp protease adaptor protein ClpS
ClpX	ATP-dependent Clp protease ATP-binding subunit ClpX
ClpXP	ClpXP protease specificity-enhancing factor
DacA	D-alanyl-D-alanine carboxypeptidase (EC 3.4.16.4)
DegQ	Outer membrane stress sensor protease DegQ, serine protease
DegS	Outer membrane stress sensor protease DegS
DP	ATP-dependent protease domain protein (EC 3.4.21.-)
IsoAsp-	Isoaspartyl aminopeptidase (EC 3.4.19.5)
LonII	ATP-dependent protease La (EC 3.4.21.53) Type II
PepA	Cytosol aminopeptidase PepA (EC 3.4.11.1)
PepB	Peptidase B (EC 3.4.11.23)
PepD	Aminoacyl-histidine dipeptidase (Peptidase D) (EC 3.4.13.3)
PepE	Alpha-aspartyl dipeptidase Peptidase E (EC 3.4.13.21)
PepN	Membrane alanine aminopeptidase N (EC 3.4.11.2)
PepP	Xaa-Pro aminopeptidase (EC 3.4.11.9)
PepQ	Xaa-Pro dipeptidase PepQ (EC 3.4.13.9)
RadA	DNA repair protein RadA
Zn-Dac	Zinc D-Ala-D-Ala carboxypeptidase (EC 3.4.17.14)

### 3.4 Antibiotic susceptibility pattern

**Fig. 6:**
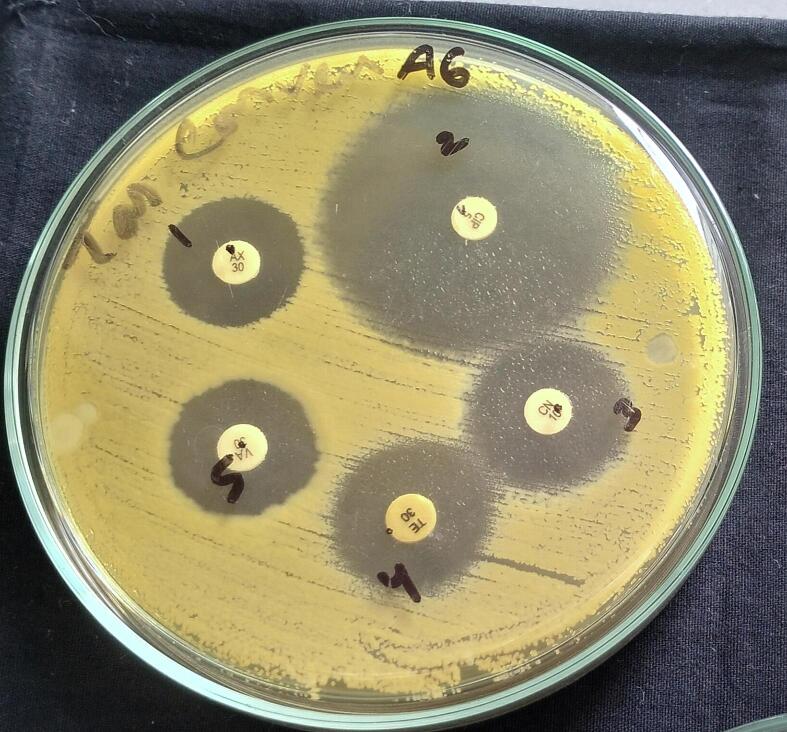
Antibiotic susceptibility test of the isolate LRI-TA6.

Since *Chryseobacterium* lacks specified criteria in CLSI, the widths of the clearance zone surrounding each disk were measured and analyzed using the performance standard of *Enterobacteriaceae* to explain the data.^30^ The study isolate LRI-TA6 was sensitive to all the five antibiotics against which it was tested with zone diameter ranges from 17.2 ± 0.8 to 32 ± 0.0 mm as shown in Table 7 and Fig. 6.

**Table 7:** Antibiotic susceptibility profile of the isolate LRI-TA6 according to CLSI guideline.

**Sl no**	**Antimicrobial agents**	**Interpretation range (mm)**				
		S	I	R	Zone diameter (mm)	Remarks
01.	Amoxicillin (30 µg)	≥18	14–17	≤13	17.3 ± 0.0	S
02.	Ciprofloxacin(30 µg)	≥21	16–20	≤15	32.0 ± 0.0	S
03.	Gentamicin(30 µg)	≥15	13–14	≤12	17.2 ± 0.8	S
04.	Tetracycline(30 µg)	≥15	12–14	≤11	18.0 ± 0.0	S
05.	Vancomycin(30 µg)	≥17	15–16	≤14	18.3 ± 0.6	S

S: sensitive; I: intermediate resistant; R: resistant.

### 3.5 Optimization of keratinase production (pH and Temperature)

**Fig. 7:**
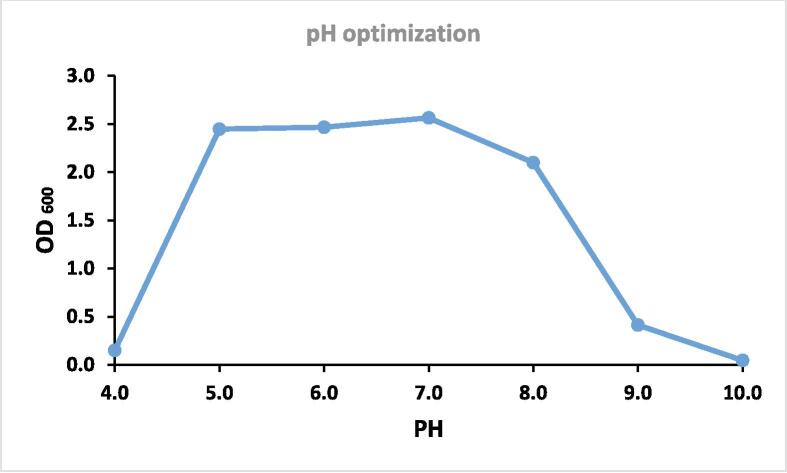
Ph optima of the isolate lri-ta6.

With an ideal growth pH of around 7.0, the isolate LRI-TA6 demonstrated growth over a wide pH range of 5.0 to 8.0 and the growth slowed down below pH 5.0 and above pH 8.0 (Fig. 7).

**Fig. 8:**
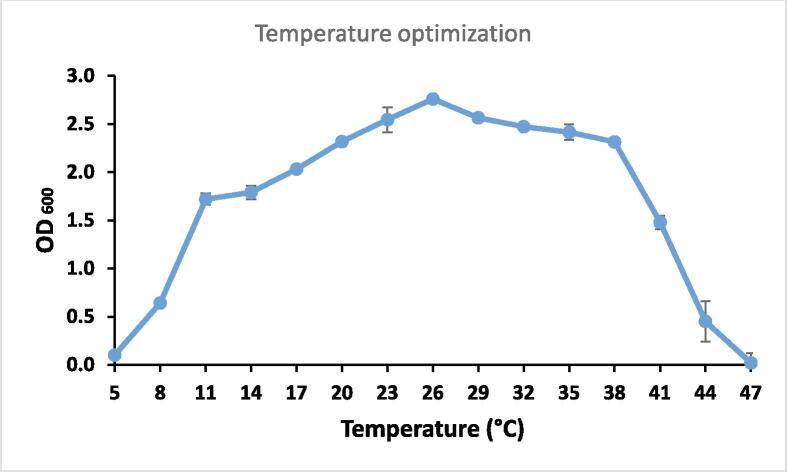
Optimum Temperature of the isolate LRI-TA6.

The isolate LRI-TA6 demonstrated enzyme production over an extended range of temperature of 10 °C to 38 °C with an optima of 25 °C and the growth abruptly slowed down below 10 °C and above 38 °C (Fig. 8).

### 3.6 Keratinase production, assay, and dehairing potency

The keratinolytic activity of the strain LRI-TA6 was determined to be 29.9 ± 6.7 and the proteolytic activity was executed to be 83.6 ± 0.2 (Table 8).

**Table 8:** Keratinolytic and proteolytic activity (U/ml) of LRI-TA6.

**Isolate**	**Keratinolytic activity (U/ml)**	**Proteolytic activity (U/ml)**
TA6	29.9 ± 6.7	83.6 ± 0.2

**Fig. 9:**
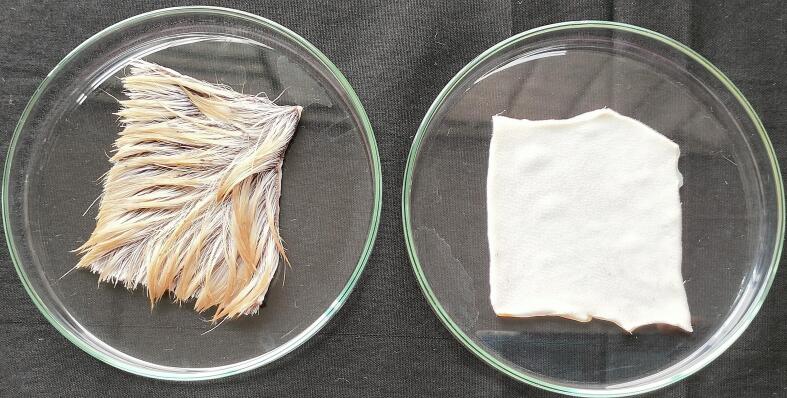
Evaluation of dehairing potency of the produced keratinase on goat skin after 18 h of incubation. The left one is control and the right one is enzyme treated dehaired goat skin.

**Fig. 10:**
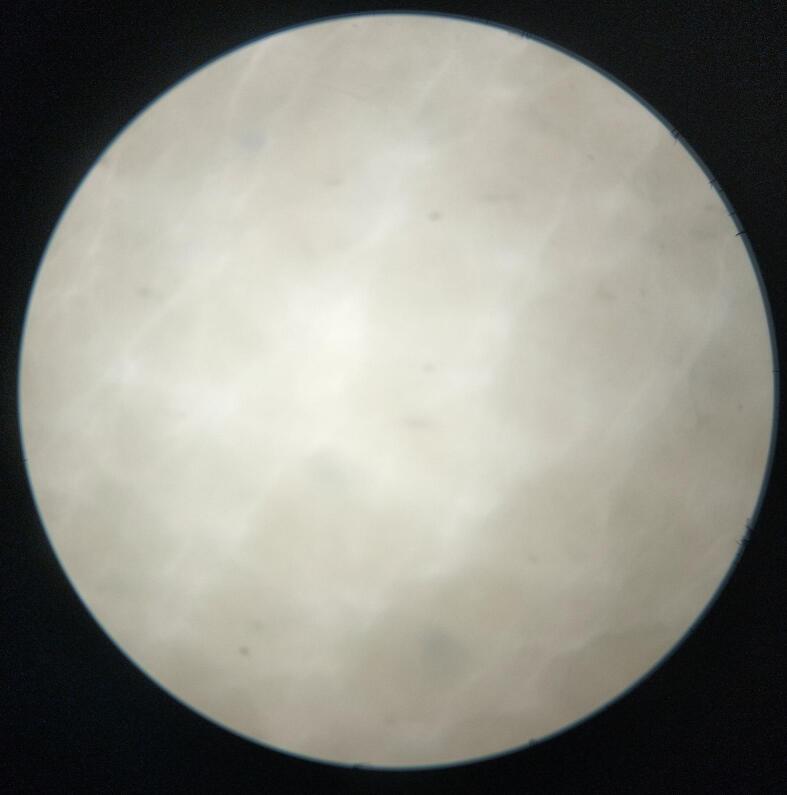

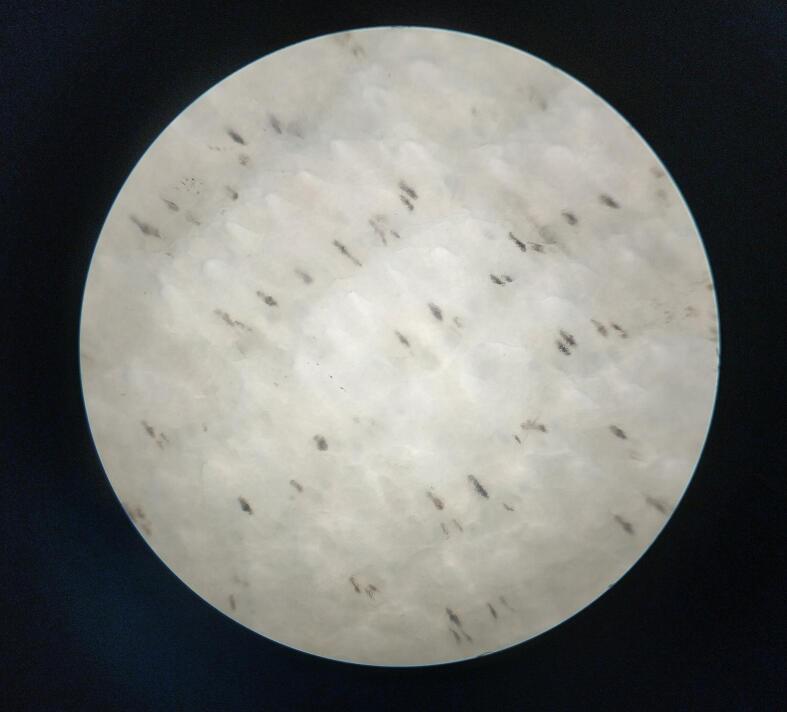
Microscopic evaluation of dehaired goat skin at 40 × magnification. (a) The skin surface dehaired with LRI-TA6 enzyme where a complete hair root removal is appeared. (b) The skin surface dehaired with LRI-TA1 enzyme with incomplete hair root removal.

At these enzymic activity, a good dehairing with smoother surface was achieved as shown in Fig. 9. The hair was completely removed from the goat skin after enzyme treatment within 18 h of incubation. To ensure hair root removal, the skin was examined microscopically with a light microscope at 40 × magnification. In case of the isolate LRI-TA1, some hair root remained after enzyme treatment but in case of LRI-TA6, there was no hair root remained instead a clean, and smooth surface was achieved as shown in Fig. 10.

## 4 Discussion

A total of ten bacteria were initially isolated from the collected sample, and based on their clearance zone on specific media, the most potent keratinase producers were selected. The strains that produced larger zones of clearance on both SMA (Table 1) and CA (Table 2) media were elected as potent keratinase producer (LRI-TA1, LRI-TA5, LRI-TA6) and finally screened on FMA media for checking their ability to utilize keratin as substrate. Of the three isolates (LRI-TA1, LRI-TA5, LRI-TA6), LRI-TA6 showed the highest zone of clearance on both SMA and CA with zone diameters of 22 ± 1.0 mm and 22.3 ± 0.6 mm (Table 1, and Table 2), respectively. LRI-TA6 also showed better growth on FMA (Fig. 1) compared to the other two (LRI-TA1, LRI-TA5) isolates. This means that the isolate LRI-TA6 can utilize keratinous feather substrate as a source of nutrients by producing the enzyme keratinase which degrades the keratin into simpler molecules.^42^ The presence of keratin in media triggers the expression of the gene responsible for keratinase production and secrete the enzyme into the media by the potent bacteria.^43^ There exists some records of keratinase synthesis and keratin degradation by *Chryseobacterium* sp.^44^, ^45^ Based on the zone diameter and growth on FMA (Fig. 1), LRI-TA6 isolate was selected for further assay.

Then, the strain LRI-TA6 was characterized both morphologically and biochemically. The bacterium was yellowish orange in color, small, circular, 3 mm colony size, entire margin, microscopically Gram-negative, non-motile, mucoid and opaque (Table 3) which correlates the findings of Jeong et al.^46^ Also, the isolate showed a positive result on catalase, oxidase, indole and MR-VP test, and also possesses the ability to hydrolyze casein and starch but couldn’t utilize citrate as a source of nutrients.^46^ However, the isolate LRI-TA6 showed some dissimilarity to the findings of Jeong et al.^46^ as it showed slight acid production on dextrose, sucrose, mannitol, and fructose fermentation test and also couldn’t hydrolyze gelatin (Table 3).

After that, we continued utilizing Illumina technology to sequence its entire genome. Initial sequencing and assembly produced genome of 4,541,898 bp in length, 36.38 % of an average GC content, and had 191 contigs as shown in Table 4. Zhao et al.^47^ reported that the genomic size of *Chryseobacterium* sp. ZHDP1, which was isolated from the soils of seafood markets, is 4,917,748 bp, and its GC content is 35.95 %. There was 4,426 protein coding sequences (CDS), 49 tRNA genes, and 3 rRNA genes in the genome of LRI-TA6 as shown in Table 4 which is quite similar to the findings of Zhao et al.^47^ To clarify the taxonomic position of LRI-TA6, according to PubMLST^47^ database, the isolate was spotted as *Chryseobacterium*. A phylogenetic study of the gene with a tree generated (Fig. 2) showed 100 % sequence homology of LRI-TA6 to the respective species *Chryseobacterium cucumeris.* According to the sequence's circular map (Fig. 3), the LRI-TA6 was arranged with a characteristic bacterial GC skew that extended from the replication initiation region to the replication termination region.

The annotation results of the genome of LRI-TA6 is shown in Table 5. There were 2,094 hypothetical proteins along with other proteins. Analysis of the subsystems which is unique to each genome was done using PATRIC (Fig. 4). Proteins that collaborate to perform a certain biological activity or structural complex are referred to as subsystems. ^48^ Out of all the proteins encoded in the strain LRI-TA6 genome, 17 % could be classified into subsystem categories and the most abundant subsystem feature counts were amino acids and derivatives (249 counts), protein metabolism (143 counts), and carbohydrates (129 counts) as shown in Fig. 4. Preliminary investigation revealed eighteen potential proteolytic enzymes in the subsystem responsible for protein degradation, given the proteolytic activity of the bacterium LRI-TA6.

Proteases are categorized into four groups: metalloproteases, aspartate proteases, cysteine proteases, and serine proteases. ^49^ Serine proteases make up around one-third of all known proteases, making them the most prevalent type of protease.^50^, ^51^ In this investigation, the 25 proteolytic gene were found to be substantially enriched for serine protease (DegQ), ATP-dependent protease DP (EC 3.4.21.-), ATP-dependent Clp protease, ATP-dependent protease, aminopeptidase, protease specificity-enhancing factor, and carboxypeptidase (Table 6). Li et al.^52^ reported proteases and peptidases producing genes in *Laceyella sacchari*. Serine proteases make up the majority of the proteases in the LRI-TA6 genome (Fig. 5), and the distribution of proteases across all *Bacillus* genomes is similar in terms of the proportion of proteases from PDB entries.^6^, ^53^ The accession number of the isolate LRI-TA6 is PRJNA1179663 which was achieved upon submission of the sequence to NCBI, SRA database. The complete genome sequence of LRI-TA6 provides insight into genetic information and the enzyme coding gene of LRI-TA6 could be employed as a possible gene source for commercial implications like dehairing of animal hide, degradation of keratinous waste, and so on.

*Chryseobacterium* sp. can be found in a variety of settings, such as rhizospheres, fish, plants, chicken, and raw milk.^46^, ^54^, ^55^, ^56^. Jeong et al.^46^ reported *C. cucumeris* from cucumber root but the present studied bacteria were obtained from a different source and that is the precipitated chrome-cake waste from tannery industries which is an addition to the source of finding this type of bacteria.

As the present study bacteria LRI-TA6 (*C. cucumeris)* is a new isolate, it’s antibiotic susceptibility pattern was demonstrated. Little research has been done on the antibiotic susceptibility pattern of *Chryseobacterium* sp. that has been isolated from ill fish or fish habitats.^57^ The majority of *Chryseobacterium* species exhibit resistance to a broad spectrum of antimicrobial agents. 57, 58 According to kim et al.^59^ *Chryseobacterium sp*. is resistant to gentamicin and ciprofloxacin which is dissimilar to our findings as we have found that the isolate LRI-TA6 (*C. cucumeris*) (isolated from tannery waste) showed sensitivity to gentamicin (17.2 ± 0.8 mm) and ciprofloxacin (32.0 ± 0.0 mm) (Table 7). Moreover, the isolate LRI-TA6 exhibited sensitivity to all five antibiotics tested (amoxicillin, tetracycline, gentamicin, ciprofloxacin, and vancomycin) as shown in Table 7 and Fig. 6. This indicates that the newly isolated strain LRI-TA6 possesses sensitivity to common antibiotics and can be used for industrial applications. However, its pathogenicity needs to be checked before commercial utilization.

The pH and temperature optima for keratinase synthesis of the identified LRI-TA6 (*C. cucumeris*) was estimated. The ideal growth pH was 7.0, while it showed growth over a wider pH range of 5.0 to 8.0 (Fig. 7). This result suggested that the study isolate LRI-TA6 is acidophilic to alkaline in its approach to keratinase synthesis and many other keratinase synthesizing bacteria with neutral to alkaline nature have been reported.^9^ The present study isolate LRI-TA6 demonstrated enzyme production over a wide temperature range of 10 °C to 38 °C (Fig. 8) which indicates its mesophilic nature and this feature is appealing from an industrial and commercial standpoint since it may yield adequate output with little energy expenditure. Keratinase synthesis by *Chryseobacterium* sp. at pH 5–6 and 25 °C has been reported by Hendrick et al.^42^ Some other studies of keratinase production at 25 °C to 28 °C are also available.^60^ From these results, it can be said that the isolated bacteria *C. cucumeris* is capable to generate enzymes throughout a larger pH and temperature range which is advantageous for its large-scale industrial implication.

Enzymatic extract of LRI-TA6 (*C. cucumeris*) manifested 29.9 ± 6.7 U/ml and 83.6 ± 0.2 U/ml of keratinolytic and proteolytic activities, respectively (Table 8). It is quite apparent that the study isolate *C. cucumeris* is a potent keratinolytic protease producer. There are also reports of keratinolytic protease enzyme production by *C. sediminis*,^61^ and *C. indologenes*. ^52^ The isolate LRI-TA6 (*C. cucumeris)* was determined to be Gram-negative. There are reports of keratinolytic protease production by Gram-negative bacteria,^11^, ^16^ especially *Chryseobacterium*^42^.

When the enzyme was applied on goat skin, a full excision of hair from the skin was achieved with a smoother surface area (Fig. 9) within 18 h. The microscopic observation of the skin showed a complete removal of hair root (Fig. 10) following LRI-TA6 enzyme treatment and it was compared with hair root removal capability of the isolate LRI-TA1 where a complete hair root removal was not achieved. The enzyme attacks the S-S bridges of the outermost sheath in the hair root and epidermis, resulting in swelling, degrading inner root sheath and causes loosening of hair^62^ and results in skin with smooth surface area^63^ as shown in Fig. 9 and Fig. 10a. Kalaikumari et al.^7^ reported that enzymatic dehairing results in good quality leather compared to conventional lime-sulfide treatment and reduce environmental contaminants. Enzymatic dehairing of hides followed by dyeing with natural dyes having antimicrobial activity^64^ might be a good way to replace hazardous chemicals.

Keratinolytic protease from different sources and having novel properties are always in demand^65^. From the above research work, it can be stated that the isolate LRI-TA6 (*Chryseobacterium cucumeris*), isolated from tannery waste, appears to be a potent candidate for keratinolytic protease production and possesses highly efficient dehairing ability as upon application on goat skin resulted in complete hair removal with smoother surface area and can be implemented, after trial on large scale and conducting additional research to purify and define the stability of the enzyme, for industrial application without causing any pollution to eco-system.

## 5 Conclusion

The above research study explores the isolation, genomic evaluation, characterization, and production of keratinolytic protease from a Gram-negative yellowish-orange bacterial strain *C. cucumeris* isolated from tannery waste sample. Very few reports have been published on the production of keratinase using *C. cucumeris* strain and to our knowledge, this reflects the first inquiry into *C. cucumeris* derived keratinase application on the dehairing process. WGS study of LRI-TA6 yielded a wealth of genomic information that served as a basis for clarifying the history of the high-yield protease-producing feature and as a helpful reference for determining the functional traits of the strain. Additionally, genome analysis provides information on the potential extracellular protease pathway and the potential use of *C. cucumeris* in the synthesis of enzymes. Our data indicates that LRI-TA6 (*C. cucumeris*) derived keratinolytic protease could be a potential green approach for leather processing industries.

**Funding**

This research is funded by Bangladesh Council of Scientific and Industrial Research (BCSIR), Dhanmondi, Dhaka 1205, Bangladesh.

## CRediT authorship contribution statement

**Taslima Akter:** Writing – original draft, Visualization, Software, Methodology, Investigation, Funding acquisition, Formal analysis, Data curation, Conceptualization. **Murshed Hasan Sarkar:** Writing – review & editing, Investigation, Formal analysis. **Shashanka Shekhar Sarker:** Investigation. **Nourin Tarannum:** Investigation. **Showti Raheel Naser:** Investigation. **Sanjana Fatema Chowdhury:** Investigation. **Sahana Parveen:** Writing – review & editing, Visualization, Validation, Supervision.

## Declaration of competing interest

The authors declare that they have no known competing financial interests or personal relationships that could have appeared to influence the work reported in this paper.
